# Plasma exosomes contain protein biomarkers valuable for the diagnosis of lung cancer

**DOI:** 10.1007/s12672-024-01022-z

**Published:** 2024-05-28

**Authors:** Zhiqiang Liu, Hong Huang, Jing Ren, Tingting Song, Yinyun Ni, Shengqiang Mao, Ying Yang, Dan Liu, Huairong Tang

**Affiliations:** 1 Department of Respiratory and Critical Care Medicine, West China Hospital, Sichuan University, Chengdu, 610041 Sichuan China; 2 Institute of Respiratory Health, Frontiers Science Center for Disease-Related Molecular Network, West China Hospital, Sichuan University, Chengdu, Sichuan China; 3 Precision Medicine Center, Precision Medicine Key Laboratory of Sichuan Province, West China Hospital, Sichuan University, Chengdu, Sichuan China; 4 Center of Health Management, West China Hospital, Sichuan University, Chengdu, 610041 Sichuan China; 5 The Research Units of West China, Chinese Academy of Medical Sciences, West China Hospital, Chengdu, Sichuan China

**Keywords:** Lung cancer diagnosis, Exosome protein, Biomarker, Proteomics

## Abstract

Accumulating evidence indicates that exosomal proteins are critical in diagnosing malignant tumors. To identify novel exosomal biomarkers for lung cancer diagnosis, we isolated plasma exosomes from 517 lung cancer patients and 168 healthy controls (NLs)—186 lung adenocarcinoma (LUAD) patients (screening (SN): 20, validation (VD): 166), 159 lung squamous carcinoma (LUSC) patients (SN: 20, VD: 139), 172 benign nodules (LUBN) patients (SN: 20, VD: 152) and 168 NLs (SN: 20, VD: 148)—and randomly assigned them to the SN or VD group. Proteomic analysis by LC–MS/MS and PRM were performed on all groups. The candidate humoral markers were evaluated and screened by a machine learning method. All selected biomarkers were identified in the VD groups. For LUAD, a 7-protein panel had AUCs of 97.9% and 87.6% in the training and test sets, respectively, and 89.5% for early LUAD. For LUSC, an 8-protein panel showed AUCs of 99.1% and 87.0% in the training and test sets and 92.3% for early LUSC. For LUAD + LUSC (LC), an 8-protein panel showed AUCs of 85.9% and 80.3% in the training and test sets and 87.1% for early LC diagnosis. The characteristics of the exosomal proteome make exosomes potential diagnostic tools.

## Highlights

- This study aimed to identify plasma exosomal proteins by proteomics for the diagnosis of lung cancer.
- Machine learning predicted more than 85% AUC in early lung cancer diagnosis.
- The diagnostic markers differences existed between LUAD and LUSC.

## Introduction

Lung cancer is the leading cause of malignancy-related mortality worldwide [1], and its 5-year survival rate is only 15% [2]. However, the 5-year survival rate of patients diagnosed at an early stage can be as high as 90%, indicating the importance of early lung cancer diagnosis [3–5]. Although low-dose computed tomography (CT) has been widely employed clinically, it was found to have a high false-positive rate. Furthermore, radiation injury and the high cost associated with CT scanning have been points of controversy [6–9]. Less-invasive techniques with high sensitivity and specificity are needed for diagnosing and monitoring early-stage lung cancer [10]. Due to their noninvasive, convenient and inexpensive acquisition methods, circulating biomarkers are a widely accepted new approach for detecting primary lung cancer and metastases [11]. Circulating biomarkers include carcinoembryonic antigen (CEA), carbohydrate antigen 19–9 (CA199), carbohydrate antigen 12–5 (CA125), cytokeratin 19 fragment (Cyfra21-1) and neuron-specific enolase (NSE). However, most of these markers are sensitive only in advanced lung cancer (stage III + IV) patients and have no benefit in the early screening of lung cancer [12]. Therefore, screening for and identifying biomarkers for the early diagnosis of lung cancer is an urgent need [10].

Liquid biopsy samples, containing mainly circulating tumor cells (CTCs), circulating tumor DNA (ctDNA) and exosomes, function well for monitoring cancer progression, relapse, and treatment effects. Among the invaluable tumor biomarkers in liquid biopsy are exosomes—lipid bilayer, nanosized (30–150 nm diameter) vesicles that are secreted into the extracellular microenvironment by almost all cell types and participate in various biological processes [13, 14]. Exosomes contain specific proteins, enzymes, metabolites, lipids and nucleic acids and are present in different fluids, such as blood, urine, saliva and ascites [15]. Exosomes derived from tumors can exchange oncogenic molecules with nearby and distant cells to establish conditions favorable for cancer growth and metastasis [16]. Due to their abundance of cancer biology-related molecules, exosomes have attracted considerable attention in tumor biomarker detection [16].

In this study, we isolated exosomes from the plasma of lung cancer patients, patients with benign lung diseases and healthy controls to identify the proteomic profile of plasma exosomes. By analyzing the protein expression differences between groups using a machine learning method, we selected the best panels for different types of lung cancer. Further receiver operating characteristic (ROC) analysis showed that these proteins had high sensitivity, specificity and area under the ROC curve (AUC) values for diagnosing early lung cancers.

## Materials and methods

### Study design and patient enrollment

**Fig. 1:**
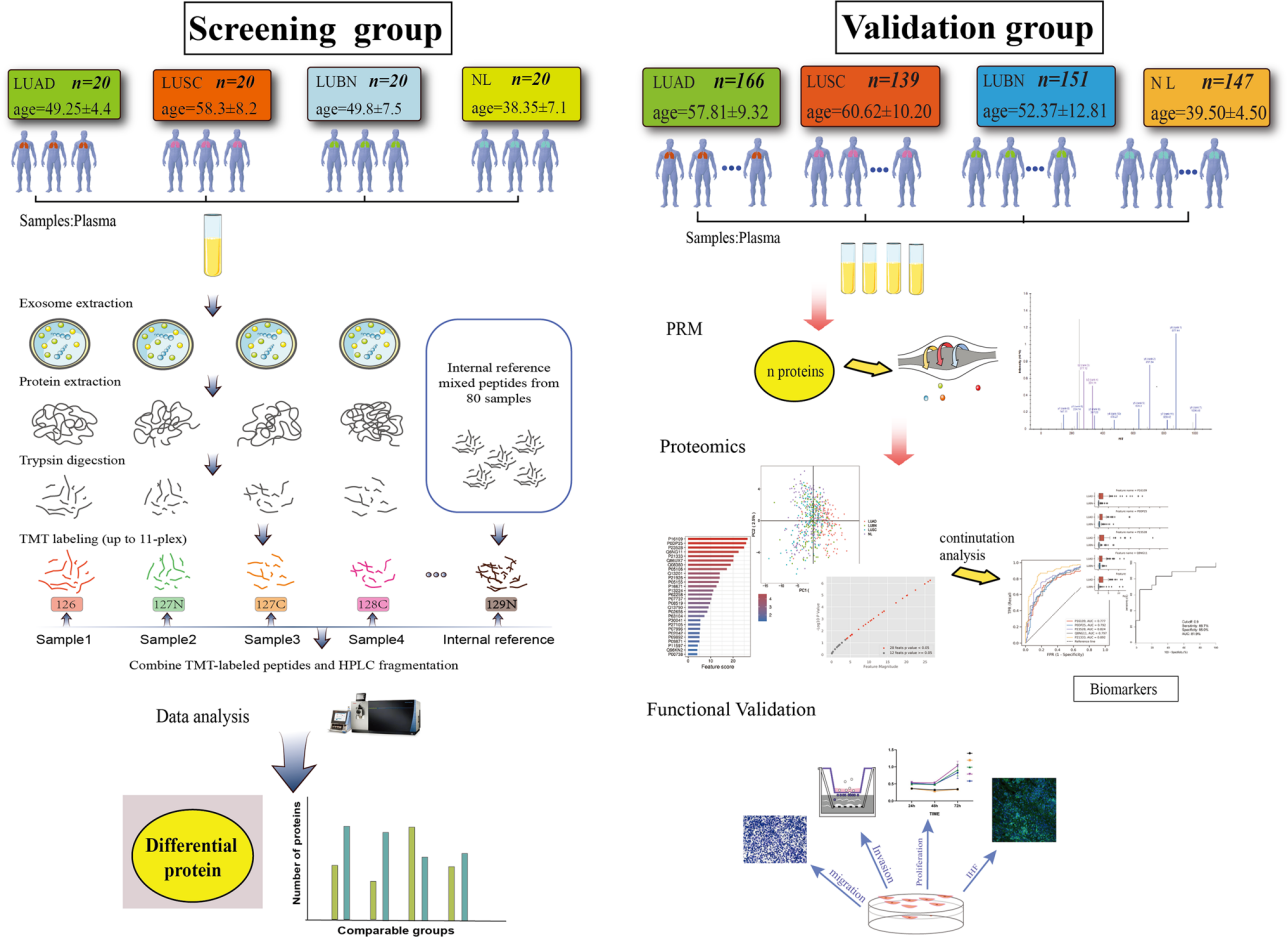
Study design. Plasma samples were randomly divided into a SN group and a VD group, and each group consisted of four plasma types (LUAD, LUSC, LUBN and NL). Screening group: Plasma samples were subjected to trypsin digestion and TMT/iTRAQ labeling, and the DEPs were screened out. Validation group: PRM and machine learning were used to verify the biomarkers, and functional verification was carried out

All 684 participants were randomly assigned to the screening (SN) or validation (VD) group: 186 lung adenocarcinoma (LUAD; ADC) patients (SN group: 20, VD group: 166), 158 lung squamous carcinoma (LUSC; SCC) patients (SN group: 20, VD group: 138), 172 benign nodule (LUBN) patients (SN group: 20, VD group: 152) and 168 healthy controls (NLs; SN group: 20, VD group: 148). Lung cancer and benign disease samples were collected from patients before therapy in the Department of Thoracic Surgery and Center of Lung Cancer, West China Hospital, between 2017 and 2020. Blood samples were collected two weeks prior to treatment, which included surgical resection, radiotherapy and chemotherapy. The NL volunteers were enrolled from the Physical Examination Centre of West China Hospital, and malignancy or benign tumors were excluded by routine physical examination results as well as the family history of tumors. All important clinical characteristics of the enrolled patients are listed in Table 1. The study design is summarized in Fig. 1.

**Table 1:** Clinical characteristics of NSCLC patients and noncancer controls

Characteristics	LUAD	LUSC	LUBN	NL
Screening group				
Age (mean ± SD)	49.25 ± 4.44	58.3 ± 8.22	49.8 ± 7.5	38.35 ± 7.13
Gender				
Male	11	19	6	4
Female	9	1	14	16
Smoking status				
Current or former	9	16	4	/
Never	11	4	16	/
Stage				
I	9	6	/	/
II	1	4	/	/
III	6	9	/	/
IV	4	0	/	/
Validation group				
Age (mean ± SD)	57.81 ± 9.32	60.62 ± 10.20	52.37 ± 12.81	39.50 ± 4.50
Gender				
Male	50	115	72	63
Female	116	23	80	80
Smoking status				
Current or former	36	95	39	/
Never	130	38	113	/
Stage				
I	148	48	/	/
II	6	37	/	/
III	5	35	/	/
IV	2	5	/	/

### Collection of plasma samples and isolation of exosomes

The study was approved by the Ethics Committee on Biomedical Research, West China Hospital of Sichuan University, following the principles of the Helsinki Declaration and obtaining the consent of all patients.Plasma was collected according to standard protocols by centrifuging whole blood with anticoagulant at 1600 ×*g* and 4 °C for 15 min and then removing the remaining red blood cells and leukocytes. The plasma was then transferred to a 1.5-mL cryopreservation tube and frozen at − 80 °C. Use the qEV Original chromatography column from Zion, which is a tool designed for extracting and purifying extracellular vesicles based on the principle of particle size exclusion. Extracellular vesicles are rapidly extracted within 15 min, removing 99% of soluble proteins, and are non-destructive, ensuring their integrity. Meanwhile, mass spectrometry was used to detect exosome marker proteins, verifying the reliability of the extraction. The isolated exosomes were dissolved in 8 M urea and extracted according to the instructions.

### Trypsin digestion

Dithiothreitol was added to the protein solution to a concentration of 5 mM and incubated at 56 °C for 30 min. Then 11 mM iodoacetamide was used and incubated at room temperature for 15 min in the absence of light. Finally, urea in the protein sample was diluted below 2 M by adding 100 mM TEAB. Trypsin was added to the sample at a mass ratio of 1:50 (trypsin:protein) and digestioned at 37 °C overnight. Then trypsin was continued to be added at a mass ratio of 1:100 (trypsin:protein) and digestioned for 4 h.

### Tandem mass tag (TMT)/isobaric tags for relative and absolute quantitation (iTRAQ) labeling

Peptide segments were labeled according to the TMT/iTRAQ kit operating instructions. The peptides were dissolved with 0.5 M TEAB, and then the defrosted labeling reagent was dissolved with acetonitrile. The peptides were mixed with the reagent and incubated at room temperature for 2 h. The labeled peptides were desalted using Strata X C18 (Phenomenex) desalt column, and then vacuum freezing-dried for use.

### High-performance liquid chromatography (HPLC) fractionation

The peptides were frayed using a high pH reverse HPLC system with Thermo Betasil C18 column (5 μm particle diameter, 10 mm inner diameter, 250 mm length). Grading gradient parameters: 8–32% acetonitrile, pH9. Under these conditions, the peptide was divided into 60 stages within 60 min, then combined into 6 components and dried by vacuum centrifuge.

### Liquid chromatography–tandem mass spectrometry (LC–MS/MS) analysis

The peptides were dissolved with 0.1% formic acid (solvent A) and isolated using the EASY-nLC 1000 ultra-high performance liquid phase system at a constant flow rate of 400nL/min. The liquid phase elution gradient is as follows: solvent B (98% acetonitrile and 0.1% formic acid) increases from 6 to 23% for 26 min, from 23 to 35% in 8 min, from 35 to 80% in 3 min, and remains at 80% in the last 3 min.

The separated peptides were ionized by injection into a nanospray ionization (NSI) source and then analyzed by MS/MS in QExactiveTM Plus (Thermo). The NSI ion source voltage is set to 2.0 kV. The full scan range is 350–1800 m/z and the resolution is 70,000. The normalized collision energy (NCE) was set to 28 and the fragment was detected at a resolution of 17,500 in Orbitrap. Automatic gain control (AGC) is set to 5E4 and the fixed first mass is set to 100 m/z.

### Database search

The Maxquant (v1.5.2.8) search engine was used to identify peptides and proteins. Select the UniProt database for a search match. The basic parameters of Maxquant (v1.5.2.8) are set to a mass fault tolerance of 20 ppm and 5 ppm for the primary and secondary search, respectively, and a mass fault tolerance of 0.02 Da for the secondary fragment. The aminomethyl of cysteine is fixed modification and acetylation modification, and the oxidation of methionine is variable modification.Using Trypsin/P, the maximum number of missed sites tolerated was set to 4, the false discovery rate (FDR) was adjusted to < 1%, and the minimum score of the modified peptide was set to > 40.

### Bioinformatics analysis

This study was based on the GO database to annotate and analyze differential expression of proteins from Cellular Component (CC), Molecular Function (MF) and Biological Process (BP). Annotation of protein pathways was done through the KEGG pathway database. InterProScan, an algorithm based on protein sequence, was used to predict the function of the protein. Wolfpsort software was used to annotate the differentially expressed proteins at the subcellular localization level. Statistically significant enrichment was determined using Fisher’s exact test, with a P value of < 0.05 corrected by Benjamini Hochberg. The clustering relationships are visualized using heat maps drawn by the function heatmap. 2 in the R language package gplot. Proteomic analysis was supported by Jingjie PTM Biological Laboratory (Hangzhou, China).

### Statistical analysis

Bioinformatic analysis of the proteome data in the VD group was performed by a machine learning method [17, 18]. Statistical significance was determined with two-tailed Student’s t-test or one-way ANOVA. P < 0.05 was considered statistically significant. The sensitivity and specificity of all biomarkers for lung cancer diagnosis were evaluated by estimating ROC curves and calculating AUCs with 95% confidence intervals (CIs). ROC curves were compared with GraphPad Prism version 8 and R software. Illustrator CC (version 2018, Adobe) was used for image editing and presentation.

### Reagents and consumables for in vitro validation

The non-small cell lung cancer (NSCLC) cell line H1299 was cultured in RPMI-1640 medium (Gibco, Grand Island, NY, USA) supplemented with 10% fetal bovine serum. All overexpression plasmids were constructed by Chengdu Yeda Technology Co., Ltd. A transfection kit (jet PRIME, 21Y0910L3) was used to transfect plasmids into cells for 48 h. Cell proliferation was detected with a Cell Counting Kit (CCK8; Zeta, Z0207205508C, Shanghai, China). Cells were seeded into Transwell chambers (8 μm, Millipore Corporation, USA) with (invasion) and without (migration) 10 mg/mL Matrigel (354,248, Corning, Jiangsu, China) precoated in 10% fetal bovine serum RPMI-1640 medium. Cells that migrated or invaded through the membrane and attached to the bottom of the Transwell plates were counted 48 h post seeding. A Cell Cycle Analysis Kit (KeyGEN BioTECH, KGA512, Jiangsu, China) and Cell Apoptosis Kit (KeyGEN BioTECH, KGA108) were used to assess the cell cycle distribution and apoptosis, respectively.

## Results

### Study design

This study aimed to identify specific exosomal biomarkers that could be applied for the early screening and diagnosis of lung cancer. For this purpose, a total of 685 patients were enrolled and divided into the screening and VD groups: 186 LUAD patients (SN group: 20, VD group: 166), 159 LUSC patients (SN group: 20, VD group: 139), 171 LUBN patients (SN group: 20, VD group: 151) and 167 NLs (SN group: 20, VD group: 147) (Fig. 1). The important clinical characteristics of all participants are listed in Table 1. To identify candidate biomarkers, proteins extracted from exosomes were trypsin digested and labeled with TMT kits. All labeled peptides were analyzed by quantitative MS, and variable biomarkers were selected by comparing the relative abundances of exosomal proteins among different groups.

Parallel reaction monitoring (PRM) was then used to analyze the VD group. All exosomal proteins were extracted, trypsin digested and subjected to LC–MS/MS analysis with a machine learning method. The candidate biomarkers were selected according to the sensitivity, specificity and AUC of each protein in the LUAD or LUSC group compared with the LUBN and NL groups. A total of 40 proteins were screened in the VD group and were identified as candidate biomarkers. A machine learning method was applied to analyze the sensitivity and specificity and to estimate the ROC curve of individual biomarkers. Proteomic analysis was then completed to evaluate the diagnostic efficiency of combinations of multiple biomarkers. Finally, we transfected plasmids expressing the top 5 candidate proteins into H1299 lung cancer cell lines to evaluate the functional influence of these proteins on the proliferation, apoptosis, cell cycle, migration and invasion of lung cancer cells (Fig. 1).

### Exosome identification and characterization

**Fig. 2:**
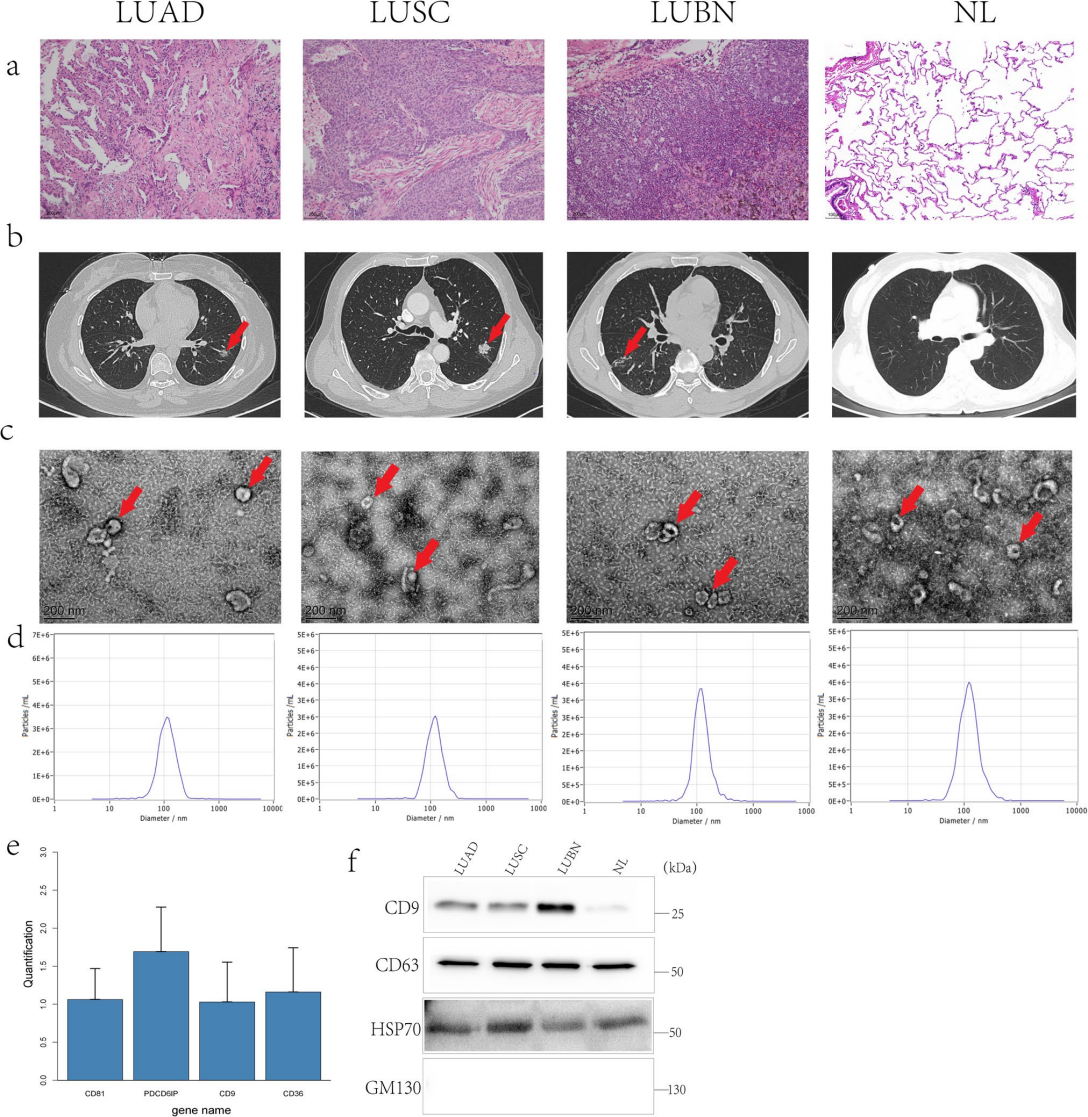
Identification and characterization of extracted exosomes. Pathological (**a**) and CT (**b**) images from randomly selected patients with LUAD, LUSC, or LUBN and NLs. TEM images of (**c**) and NTA (**d**) results for plasma exosomes. **e** Typical exosomal proteins CD81, PDCD6IP, CD9 and CD36 were validated by MS. **f** Detection of exosomal positive protein markers CD9, CD63, HSP70, and exosomal negative protein marker GM130 by Western blotting.(The blotting membrane is customized based on the specific molecular weight)

In this study, LUAD, LUSC and LUBN were diagnosed by both CT and immunohistochemical staining. Representative CT and pathological images of patients with LUAD, LUSC, or LUBN and healthy controls are shown in Fig. 2a and b. Exosomes isolated from the plasma of all enrolled individuals were characterized by transmission electron microscopy (TEM), nanoparticle tracking analysis (NTA), protein profiling and Western blotting [19, 20]. Among these approaches, TEM is the gold standard for determining the presence of exosomes. Our results indicated that exosomes in all groups were dish- or cup-like vesicles with a diameter of 50–100 nm and a lipid bilayer. During the freezing and rewarming process of plasma at – 80 ℃, large extracellular vesicles will rupture, forming small cell membrane fragment structures, leading to background differences (Fig. 2c). NTA also indicated that the average diameter of the exosomes in all groups was 100 nm (Fig. 2d), consistent with a previous study on exosome analysis (119–21). CD81, PDCD6IP, CD9 and CD36 were present in exosomes, as determined by MS [21–23] (Fig. 2e). Finally, Western blotting was performed to detect exosomal biomarkers (CD9, HSP70, CD63 and GM130) in 4 randomly selected patients. CD9 was highly expressed in 3 patients, and CD63 and HSP70 were highly expressed in all 4 patients. Exosome negative protein was not expressed (Fig. 2f). These results confirmed the specific characteristics of exosomes from all enrolled individuals.

### Functional enrichment of differentially quantified proteins and clustering for protein groups

**Fig. 3:**
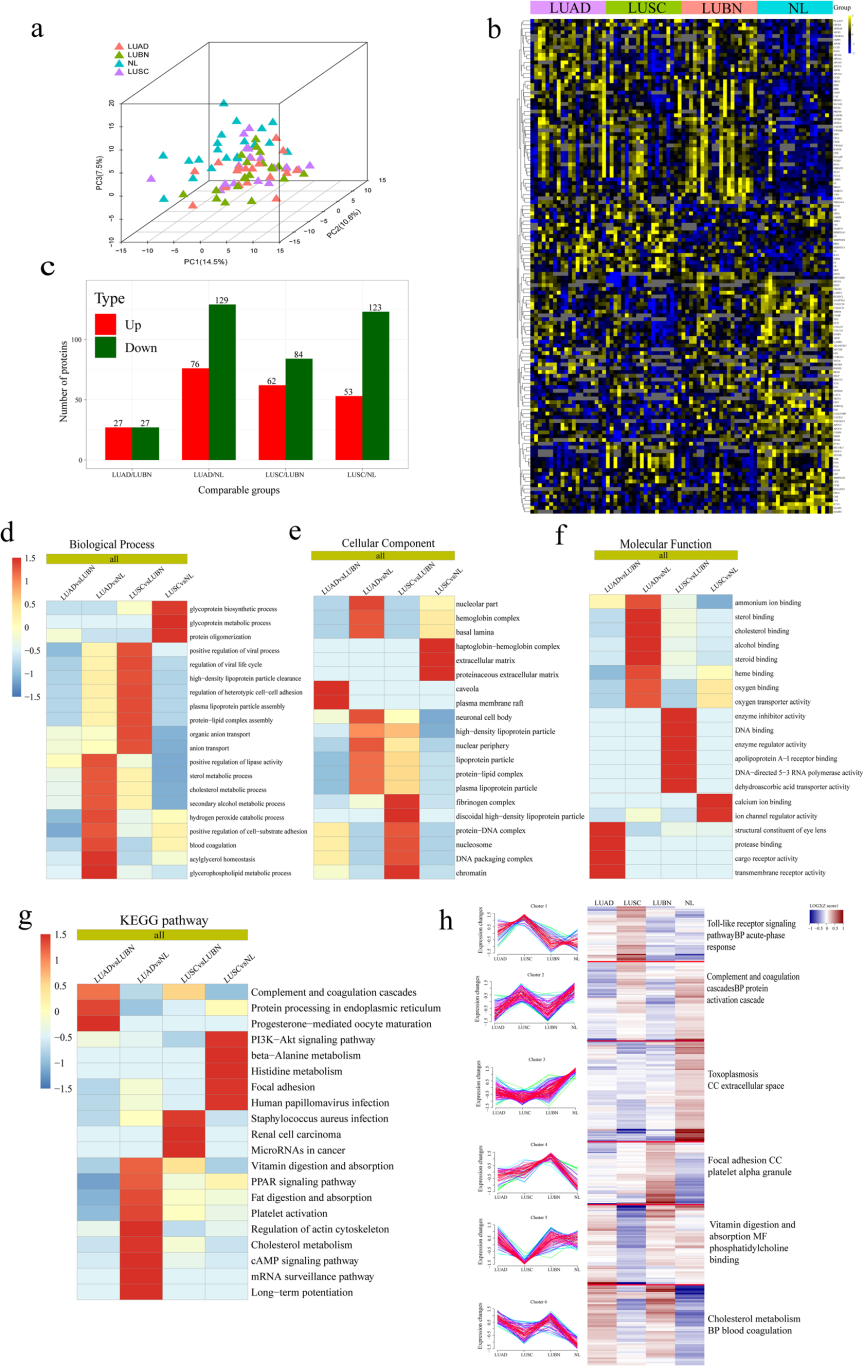
Screening differentially quantified proteins of exosomes from all groups. **a** Three-dimensional PCA of protein expression in all groups. **b** Cluster analysis of DEPs. **c** Numbers of upregulated and downregulated DEPs in the LUAD, LUSC, LUBN and NL groups (1.2-fold change, P ≤ 0.05). Hierarchical clustering analysis was conducted for the DEPs according to biological process (**d**), cellular component (**e**), molecular function (**f**), KEGG pathway (**g**) and mfuzzy c-means algorithm-based enrichment (**h**). P values were transformed into Z-scores for hierarchical clustering analysis. The Z-scores are shown in the color legend. Red indicates significant enrichments

A total of 1403 proteins were identified, and 1059 proteins were quantified with one or more unique peptides. To demonstrate the general pattern of protein abundance variation among the different groups, a three-dimensional principal component analysis (PCA) was performed based on the quantified proteins. The results confirmed that these proteins showed obvious separation between the LUAD, LUSC and NL groups, while no obvious differences were observed between the LUAD, LUSC and LUBN groups (Fig. 3a). Cluster analysis indicated that multiple proteins were differentially expressed between LUAD and NL groups and between the LUSC and NL groups (Fig. 3b) (P < 0.05, 1.5-fold up- or downregulation). A total of 17 and 42 proteins were upregulated and 14 and 87 were downregulated in LUAD patients compared with LUBN patients and NL, respectively; in contrast; 33 and 27 proteins were upregulated and 48 and 73 were downregulated in LUSC patients compared with LUBN patients and NLs, respectively (Fig. 3c). In addition, 35 proteins were upregulated and 30 were downregulated in the LUAD group compared to the LUSC group (Fig. 3c).

Regarding biological processes, GO terms related to lipid metabolic processes, including lipase activity, sterol and cholesterol, were highly enriched in the LUAD group compared with the NL group, while in the LUSC group compared with the NL group, the enriched pathways were concentrated in glycoprotein metabolic processes (Fig. 3d). Regarding cellular components, the main GO terms highly enriched in the LUAD compared with the NL group were nucleolus, hemoglobin complex, and nucleolar part; the terms enriched in the LUSC compared with the NL group were haptoglobin-hemoglobin complex and extracellular matrix pathways (Fig. 3e). We next analyzed the molecular functions. The main GO terms highly enriched in the LUAD compared with the NL group were oxygen binding, oxygen transporter activity, substrate-specific transporter activity, lipase binding and other pathways, and those highly enriched in the LUSC compared with the NL group were concentrated mainly in calcium ion binding and ion channel regulator activity (Fig. 3f). In the KEGG pathway enrichment analysis, the KEGG terms highly enriched in the LUAD compared with the NL group were involved mainly in cholesterol metabolism, fat digestion and absorption, vitamin digestion and absorption, and the PPAR signaling pathway; those highly enriched in the LUSC compared with the NL group were involved mainly in beta-alanine metabolism, histidine metabolism, arginine and proline metabolism and other pathways (Fig. 3g). The abundance characteristics of the DEPs were analyzed based on the mfuzzy method and were divided into six clusters named as follows: Toll-like receptor signaling pathway BP acute-phase response, complement and coagulation cascades BP protein activation cascade, toxoplasmosis CC extracellular space, focal adhesion CC platelet alpha granule, vitamin digestion and absorption MF phosphatidylcholine binding, and cholesterol metabolism BP blood coagulation. The LUAD, LUSC, LUBN, and NL groups showed significant differences in each cluster (Fig. 3h).

In summary, our study identified multiple proteins that were significantly differentially expressed in each group. These proteins belonged to distinct pathways and were selected as candidate biomarkers for further exploration.

### Validation of exosomal biomarkers by targeted proteomic analysis

**Fig. 4:**
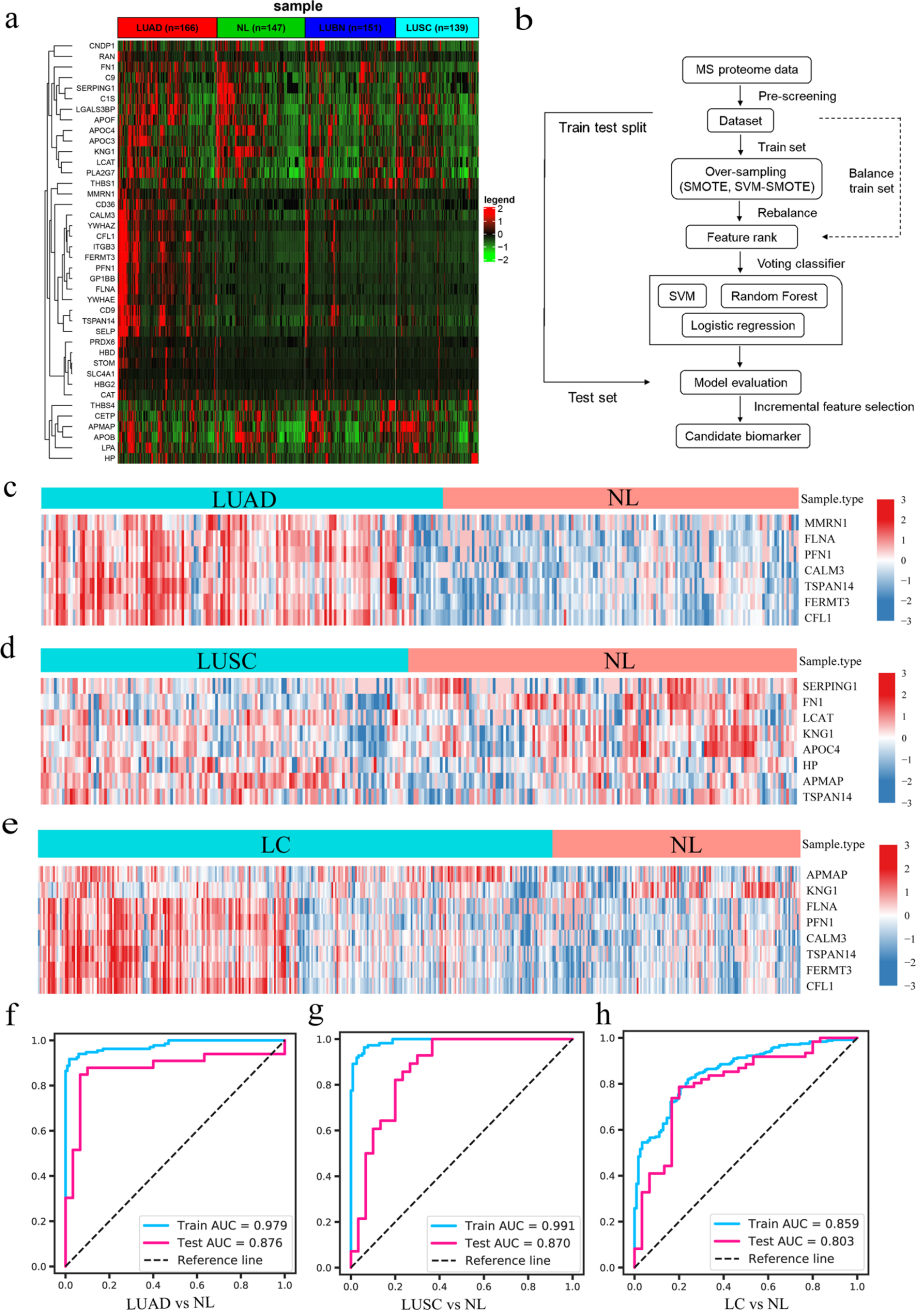
Machine learning method to verify markers. **a** PRM was used to quantify the 40 selected target proteins in the VD groups (LUAD = 166, LUSC = 139, LUBN = 151, and NL = 147). **b** Technical flowchart of the machine learning method for biomarker screening. **c**–**e** Optimal expression of local proteins, as evaluated by machine learning, in different groups. **f**–**h** The ROC curves for the training set and the test set were obtained with the optimal expression feature subset

Based on the PRM results and functional alterations revealed in the screening study, we then sought to explore exosomal protein biomarkers that could be used for the diagnosis of lung cancer. We performed PRM quantification of the selected 40 target proteins in a large cohort of 605 samples: 166 LUAD, 139 LUSC, 151 LUBN and 147 NL samples. According to cluster analysis, there were many DEPs between LUAD and NL and between LUSC and NL (Fig. 4a).

Based on the above data, we applied machine learning methods to evaluate and screen candidate exosomal protein markers. The above screening results provided direction for further in-depth analysis and confirmation of the biological functions and mechanisms of the candidate liquid biopsy exosomal markers. Through a combination of feature selection methods, machine learning algorithms, classifier ensemble methods and dataset verification, the candidate exosome markers were screened and verified (Fig. 4b).

The optimal protein expression heatmap better reflects the change pattern of the selected proteins in different samples. To screen for effective diagnostic markers for lung cancer, we searched for potential markers differentiating the LUAD and NL groups, LUSC and NL groups, and LC and NL groups. To select optimal biomarkers from the set of candidate proteins, a machine learning model was introduced to select proteins with the maximum Matthews coefficient. Based on this criterion, 7 (MMRN1, FLNA, PFN1, CALM3, TSPAN14, FERMT3 and CFL1) and 8 (SERPING1, FN1, LCAT, KNG1, APOC4, HP, APMAP and TSPAN14) biomarkers were selected for the LUAD (Fig. 4c) and LUSC groups (Fig. 4d), respectively, and 8 proteins (APMAP, KNG1, FLNA, PFN1, CALM3, TSPAN14, FERMT3 and CFL1) were selected for the LC group (Fig. 4e).

Finally, we constructed combinatorial analysis models for the different groups. In the LUAD group, the training set contained 250 samples and the test set contained 63 samples, with AUCs of 0.979 and 0.876, respectively (Fig. 4f). In the LUSC combinatorial analysis, the training set contained 228 samples and the test set contained 58 samples, with AUCs of 0.991 and 0.870, respectively (Fig. 4g). In the LC group, the training and test sets contained 361 and 91 samples, respectively, with AUCs of 0.859 in the training set and 0.803 in the test set (Fig. 4h).

Taken together, these findings indicated that we identified specific panels of exosomal proteins for LUAD, LUSC and LC.

### Panel for early diagnosis of lung cancer

**Fig. 5:**
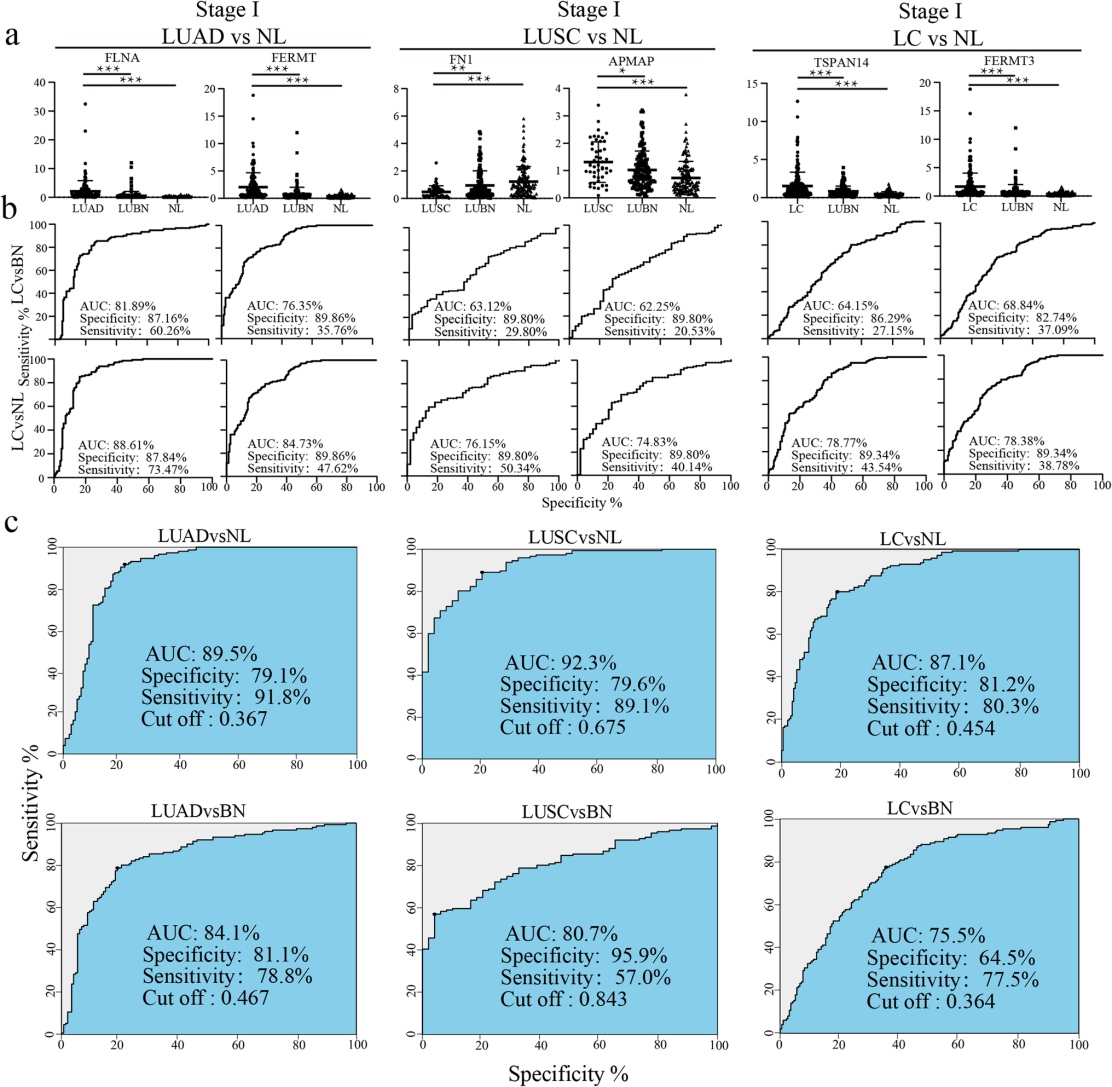
ROC analysis of optimal markers. In the optimal protein expression subsets of LUAD vs. NL, LUSC vs. NL, and LC vs. NL, the two proteins with the highest AUCs were selected for analysis of stage I lung cancer samples. **a** The expression of two biomarker proteins in each stage I sample and LUBN and NL samples (***P < 0.001). **b** ROC analysis of 2 proteins between each stage I sample and LUBN and NL samples. **c** The optimal protein combination for distinguishing between each pair of groups was used to estimate the ROC curve between each phase I group and the LUBN and NL groups

Based on use of the optimal expression biomarker panels identified by the machine learning method as the diagnostic panels, we selected early lung cancer samples (stage I) for ROC analysis. We selected the two factors with the highest AUC in each panel to differentiate between early lung cancer samples and LUBN and NL samples (Fig. 5a, b).

For LUAD, we identified the 7 optimal proteins from 166 patients with LUAD vs. 147 NLs as diagnostic markers, with combined AUCs of 89.5% for the diagnosis of 148 patients with early LUAD (stage I) and 84.1% for differentiating between early LUAD and LUBN (Fig. 5c).

For LUSC, we identified the 8 optimal proteins from 139 patients with LUSC vs. 147 NLs as diagnostic markers. The combined AUCs were 92.3% for the diagnosis of 49 early LUSC (stage I) and 80.7% for differentiating between early LUSC and LUBN (Fig. 5c).

For the total LC cohort, we identified the 8 optimal proteins from 305 patients with LC vs. 147 NLs as diagnostic markers of LC. The combined AUCs were 87.1% for the diagnosis of early LC (stage I) and 75.5% for differentiating between early LC and LUBN (Fig. 5c).

The above data show that there were significant differences in exosomal proteins between lung cancer patients and healthy controls. The panels that we identified had high sensitivity and specificity and can be used to distinguish patients with lung cancer from healthy controls.

### Functional validation in vitro

We selected the 5 proteins (TSPAN14, CALM3, FLNA, FERMT3 and APMAP) with the lowest P values in the LC vs. NL group comparison for in vitro verification.

**Fig. 6:**
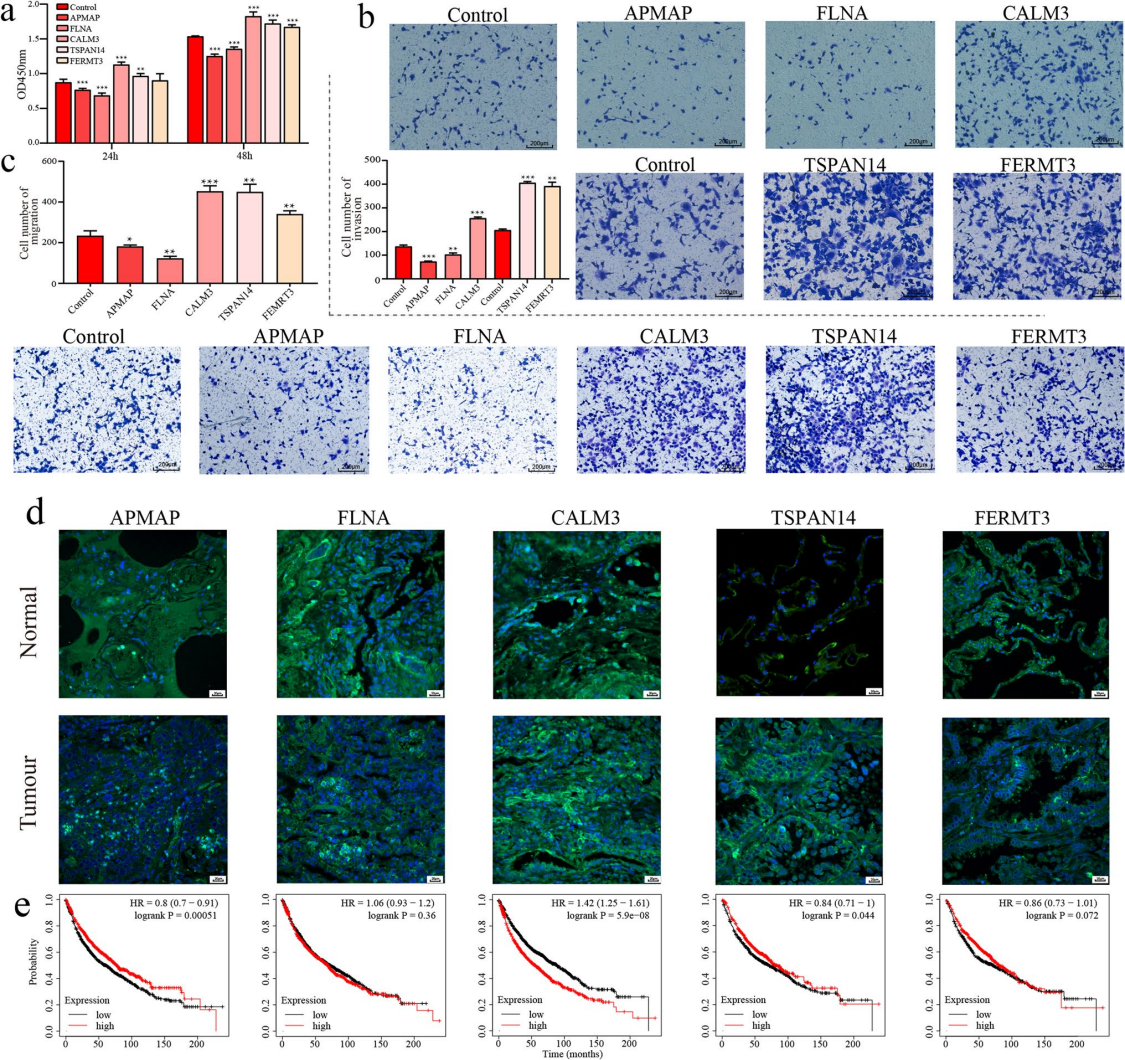
Functional verification in vitro. **a** Proliferation of H1299 cells after overexpression of each of the five selected proteins for 48 h. Invasion (**b**) and migration (**c**) of H1299 cells after overexpression of each of the five selected proteins for 2 days. The relationship between survival and the expression of each of the five selected proteins in lung cancer (**d**) and normal tissues as determined by immunofluorescence was verified with KMPlotter (**e**)

We overexpressed these proteins in H1299 cells to investigate whether the cell phenotypes changed significantly. The proliferation assay showed that the proliferation ability decreased with overexpression of APMAP and FLNA but increased with overexpression of CALM3, TSPAN14 and FERMT3 (Fig. 6a). The same trend was reflected in the migration and invasion abilities (Fig. 6b, c). Immunofluorescence staining showed that these factors are also expressed in tissues and can be detected (Fig. 6d). According to KMPlotter database analysis, APMAP and CALM3 were differentially expressed in tumors (Fig. 6e).

These data suggest that factors derived from plasma exosomes of patients with lung cancer play roles in these phenotypes.

## Discussion

Liquid biopsy has good application prospects for early cancer detection, tumor classification and treatment response monitoring. The billions of exosomes circulating in body fluids may be important components of liquid biopsy samples [24–28].

Here, we analyzed the plasma exosomal proteome of 684 human cancer and healthy control samples. Machine learning analysis showed that the differential protein expression levels in LUAD were significantly higher than those in LUSC in the comparison between exosomes derived from patients with lung cancer and healthy controls.

Independent analysis showed that the diagnostic efficacy was better for LUAD than for LUSC and LC. Proteins involved in the cell cycle, cytoskeleton, membrane, cell adhesion, signal transduction, cell movement, and actin signaling pathways were enriched in LUAD-derived exosomes, whereas proteins involved in coagulation, complement, oxidative stress, and metabolic pathways were enriched in LUSC-derived exosomes. This difference may be related to the degree of tumor differentiation and tumor heterogeneity [29, 30]. Most of the patients in the LUAD group had stage I lung cancer, but only one-third of the patients in the LUSC group had stage I lung cancer (Fig. 5c).Individual differences lead to differences in the quantity and composition of plasma exosomes in patients with different pathological subtypes of lung cancer. Plasma exosomes are specifically secreted at different stages of lung cancer, and the number and composition of exosomes vary in different stages of lung cancer.Exosomes can reflect the systemic effects of cancer on the tumor microenvironment, distant organs and immune system. [28] Therefore, minimally invasive diagnosis of asymptomatic cancer by plasma exosome markers has great clinical potential. [31] To date, there have been many studies on the secretory markers of lung cancer.

Plasma exosomal miR-200b-5p, miR-378a, miR-139-5p and miR-379 can distinguish nodule from nonnodule tissue with 97.5% sensitivity and 72.0% specificity, and exosomal miR-629, miR-30a-3p, miR-100, miR-200b-5p, miR-154-3p and miR-151a-5p can distinguish granulomas from LUAD with 96.0% sensitivity and 60.0% specificity [32]. Exosomal miR‐19‐3p, miR‐21‐5p and miR‐221‐3p can distinguish patients with LUAD from healthy controls with sensitivity and specificity ranging from 67–73% and 66–80%, respectively [33]. In addition, exosomal miR‐96 could have diagnostic value in lung cancer patients [34].

Lipopolysaccharide binding protein (LBP) in serum exosomes can distinguish patients with metastatic NSCLC from those with nonmetastatic NSCLC. ROC curve analysis showed that exosomal LBP had an AUC of 0.803 with a specificity of 67% and a sensitivity of 83.1% [35]. Four exosome-associated proteins—HUWE1, TPM3, SRGN, and THBS1—differentiated patients with LUAD from controls (AUC: 0.90) [36]. In addition, by combining a variety of protein extracellular vesicle (EV) arrays for lung cancer, two groups of healthy controls and lung cancer patients were successfully distinguished with an accuracy rate of 75.3% [37].

Compared with traditional biomarkers, exosomal proteins possess unique features, and exosomal proteins have unique characteristics [38]. First, exosomal proteins have higher sensitivity than proteins directly detected in blood [39]. Second, exosomal proteins have higher specificity than secreted proteins [40]. Third, exosomal proteins are highly stable [39]. Tumors are highly heterogeneous; thus, opportunities to identify a single diagnostic biomarker are likely few.

We evaluated more samples and more types of plasma samples than did other biomarker studies, and both the specificity and sensitivity of the identified biomarkers were greater than 80%. Moreover, we followed up all lung cancer patients. More than 90% of the patients were still alive, showing a basis for the use of our panel as a diagnostic marker for early lung cancer and indicating that the expression of the proteins in our panel may be related to the survival rate of patients. Overall, these exosomal biomarkers are differentially expressed in patients with lung cancer compared to healthy controls and are of great diagnostic value in lung cancer. The results suggest that these panels could be useful in early clinical diagnosis.

## Data Availability

The datasets generated and/or analyzed during the current research process can be provided at the reasonable request of the corresponding author.
